# *NLRP12* and *IL36RN* mutations in a Portuguese woman with autoinflammatory syndrome

**DOI:** 10.1016/j.jdcr.2022.06.011

**Published:** 2022-06-30

**Authors:** Sofia Antunes-Duarte, Ana Marcos-Pinto, Lars E. French, Heinz Kutzner, Luís Soares-de-Almeida

**Affiliations:** a Dermatology Department, Hospital de Santa Maria, Centro Hospitalar Universitário Lisboa Norte, E.P.E., Lisbon, Portugal; b Dermatology Department, LMU Hautklinik Dermatologie, Muenchen, Germany; c Dr. Philip Frost Department of Dermatology and Cutaneous Surgery, University of Miami Miller School of Medicine, Miami, Florida; d Dermatology Department, Dermatopathologie Friedrichshafen, Friedrichshafen, Germany; e Unit of Research in Dermatology, Institute of Molecular Medicine, Faculty of Medicine, University of Lisbon, Lisbon, Portugal

**Keywords:** deficiency of IL-36-receptor antagonist, familial cold autoinflammatory syndrome, IL36RN, NLRP12, systemic autoinflammatory diseases, AID, autoinflammatory disease, FCAS-2, familial cold urticaria syndrome-2, GPP, generalized pustular psoriasis

## Introduction

Autoinflammatory diseases (AIDs) encompass a genetically heterogeneous group of diseases driven by abnormal activation of the innate immune system.^1^^,^^2^ Patients share recurrent flares of fever, elevation of acute phase reactants, and variable clinical manifestations, including a wide range of cutaneous lesions.^2^^,^^3^

Familial cold urticaria syndrome-2 (FCAS-2), caused by *NLRP12* mutations, belongs to the group of cryopyrin-associated periodic syndrome and is characterized by urticarial skin lesions, while patients with deficiency of IL-36-receptor antagonist have mutations in *IL36RN* and typically present with generalized pustular psoriasis (GPP).^3^, ^4^, ^5^

We report the case of a woman with autoinflammatory syndrome who was found to harbor simultaneously mutations in the *NLRP12* and *IL36RN* genes.

## Case report

The patient is a 35-year-old Portuguese woman, apparently healthy until the age of 30 years (2012), when she developed symmetrical inflammatory polyarthralgia, with morning stiffness. She was initially treated with nonsteroidal anti-inflammatory drugs, with partial clinical response. A complete autoimmunity panel, including antinuclear antibodies and rheumatoid factor, was negative, and prednisolone 10 mg/day was empirically started with good control of the disease.

In 2016, she was hospitalized for a disseminated dermatosis characterized by erythematous papules of the extremities, knees, and elbows, accompanied by fatigue, fever, and worsening of the arthralgias. This was presumptively diagnosed as vasculitis (without cutaneous biopsy) and successfully treated by increasing the dosage of oral steroids.

**Fig 1:**
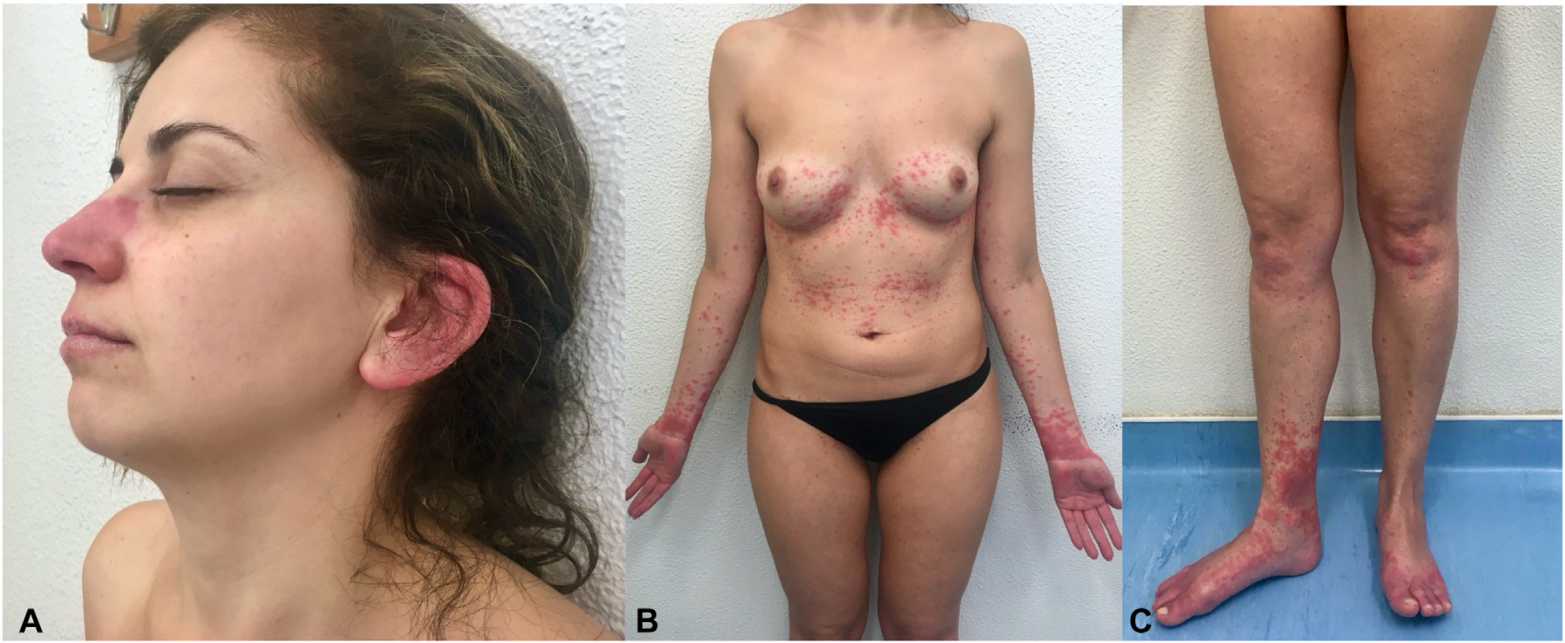
Portuguese woman with *NLRP12* and *IL36RN* mutations, clinical presentation at admission: Tumid well-demarcated erythematous papules and plaques on the nose, ears (**A**), trunk, arms, hands (**B**), legs and feet (**C**).

**Fig 2:**
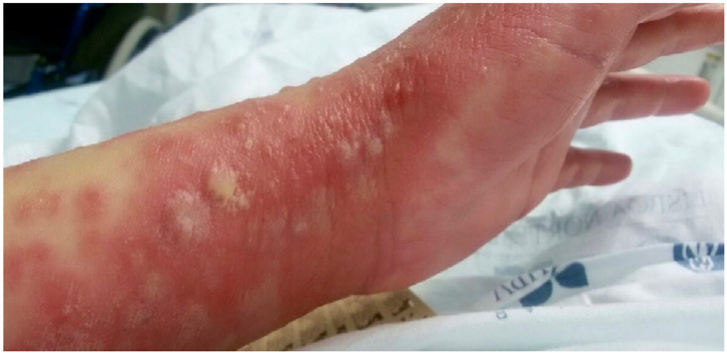
Portuguese woman with *NLRP12* and *IL36RN* mutations, clinical characteristics 1 week after admission: Multiple coalescing pustular lesions on an erythematous base.

In 2017, she was admitted to our dermatology department for a second more severe episode, which started 1 week earlier, consisting of painful well-delimited erythematous papules and plaques on the nose, ears, abdomen, forearms, hands, legs, and feet (Fig 1), along with fever (39 °C), polyarthralgia, malaise, nausea, and headache. One week after the admission, the patient developed a generalized sterile pustular eruption on an erythematous background, affecting approximately 60% of the body surface (Fig 2).

Her past medical history was remarkable for recurrent infections (tonsillitis, pharyngitis, bronchopneumonia, and viral infections due to Flu A, herpes simplex, and herpes zoster), 3 spontaneous miscarriages, and Raynaud phenomenon. There was no report of similar symptoms in her family.

On admission, laboratory data revealed leukocytosis with neutrophilia (20.460/mm^3^ white blood cells, 17.000/mm^3^ neutrophils) and elevated C-reactive protein (6 mg/dL). Serological markers for systemic autoimmune diseases, including antinuclear antibodies, antineutrophil cytoplasmic antibodies, and rheumatoid factor, were negative. Cryoglobulin and complement levels were within normal limits.

**Fig 3:**
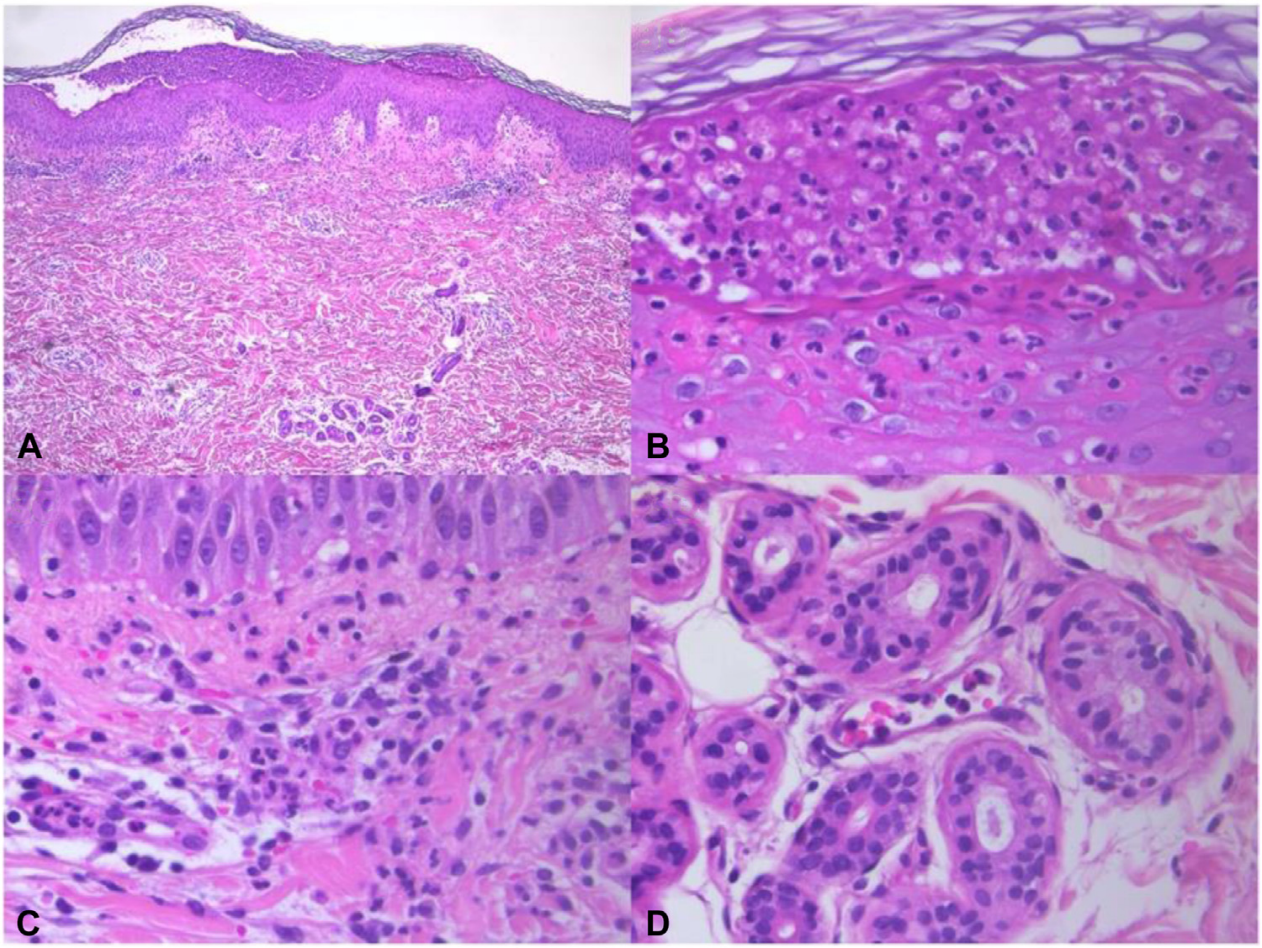
Portuguese woman with *NLRP12* and *IL36RN* mutations, histopathological findings: Combination of a neutrophilic urticarial dermatosis- and pustular psoriasis-pattern of cutaneous inflammation (**A**). Irregular acanthosis of the dermis with exocytosis of neutrophils and formation of intradermal and subcorneal spongiotic pustules (**B**). In the superficial and mid-dermis, dense neutrophilic inflammatory infiltrate with some eosinophils, located interstitially (**C**), intravascularly, and around the sweat glands (**D**). Vasculitis is not seen. (Hematoxylin and eosin, **A** - ×40; **B-D** - ×400).

Skin biopsies revealed a combination of pustular psoriasis and neutrophilic urticarial dermatosis patterns, as depicted in Fig 3. The patient was again treated with prednisolone 20 mg/day (0.3 mg/kg/day), with a good clinical response.

At this stage, an AID was evoked, and genetic analysis was thus performed using whole-exome sequencing of a panel of 13 genes. This revealed the presence of 2 heterozygous mutations of the *NLRP12* gene and 1 heterozygous variant of the *IL36RN* gene (Table I). Bioinformatic analysis of these 3 mutations identified them as likely pathogenic.

**Table I:** Genetic test: identified mutations of *IL36RN* and *NLRP12* genes in a panel of 13 genes associated with autoinflammatory diseases (*CARD14, ELANE, IL36RN, LPIN2, MEV, MVK, NLRP12, NLRP3, NOD2, PSMB8, PSTPIP1, TNFAIP3, TNFRSF1A*)

Gene	Location	Nucleotide substitution	Aminoacid substitution	Mutation type
*IL36RN* (NM_001277126)	Exon 4	c.227C>T	p.Pro76Leu	Missense
*NLRP12* (NM_012275.2)	Exon 3	c.625G>A	p.Ala218Thr	Missense
		c.910C>T	p.His304Tyr	Missense

Given the clinical, histopathologic, and genetic findings, we established the diagnosis of FCAS-2 and GPP in association with *IL36RN* heterozygous mutation. Prednisolone was gradually tapered down for 3 months, with new flare of cutaneous and articular symptoms after suspension. Since then, she has been asymptomatic with prednisolone 5 mg/day.

## Discussion

Familial cold autoinflammatory syndrome-2 (OMIM #611762) is a rare autosomal dominant disease associated with mutations in the *NLRP12* gene (band 19q13.42).^1^ This gene encodes for NLRP12 protein, a member of the larger family of NOD-like receptor, which acts as a negative regulator of inflammation, suppressing nuclear factor-κB activation and subsequent production of proinflammatory cytokines and chemokines, such as interleukin (IL)-1, IL-6, and IL-8.^1^^,^^4^^,^^6^ Since the first report of *NLRP12* as a causative gene of AIDs, at least 62 cases of FCAS-2 have been discovered worldwide.^6^ Our patient carried simultaneously 2 mutations: c.625G>A (p.Ala218Thr) and c.910C>T (p.His304Tyr). The age at onset of FCAS-2 is variable, ranging from the first year of life to middle age, with a slight male prevalence.^4^ Most of the patients are Caucasians, and approximately 50% have a positive family history.^1^^,^^4^ Although the severity and clinical manifestations are heterogeneous, patients typically present with periodic urticaria-like rash, arthralgia/arthritis, myalgia, and lymphadenopathy, accompanied by fever.^1^^,^^4^ Most patients report exposure to cold as a trigger for the episodes (66%).^1^ Though management of FCAS-2 is not standardized, glucocorticoids, antihistamine drugs, and nonsteroidal anti-inflammatory drugs have been reported to be largely effective.^1^^,^^4^ Therapy with antagonists of tumor necrosis factor-a (infliximab, adalimumab) or IL-1b (anakinra and canakinumab) has also been associated with variable results.^4^

On the other hand, deficiency of IL-36-receptor antagonist (OMIM #614204) is a rare AID caused by recessively inherited mutations in *IL36RN* (band 2q14.1).^3^^,^^5^ *IL36RN* encodes for the IL-36 receptor antagonist, a competitive inhibitor of IL-36 receptor.^5^ Loss of functional IL-36 receptor antagonist leads to unrestrained IL-36 activity, which activates nuclear factor-κB and mitogen-activated protein kinase pathways, leading to overexpression of proinflammatory cytokines, including IL-8, responsible for neutrophilic inflammation.^5^ Clinically, it is characterized by recurrent episodes of GPP associated with systemic symptoms, which can be life-threatening.^3^^,^^5^ Although deficiency of IL-36-receptor antagonist is a recessively inherited disorder, very few reports suggest that heterozygous *IL36RN* mutation may also contribute to the development of GPP, as in our patient, with the heterozygous mutation c.227C>T (p.Pro76Leu), already reported and described as pathogenic.^7^ In addition, the IL-36 receptor inhibitor spesolimab has recently been shown to induce rapid disease clearance in over 50% of patients with GPP.^8^

We present a case of a patient with recurrent polyarthralgia who had 2 flares of skin eruptions associated with fever, systemic symptoms, elevation of acute phase reactants, histological evidence of neutrophilic urticarial dermatosis, and a great response to steroid treatment. These findings are consistent with FCAS-2 phenotype, and it was genetically corroborated. Of interest, although our patient denied cold exposure as a trigger, the initial skin lesions were predominantly located on acral sites, where the temperature is lower. *IL36RN* mutation had probably contributed to the evolution of clinical presentation, characterized by a subsequent generalized pustular eruption with histopathologic features of pustular psoriasis. Taken together, we hypothesize that the full-blown phenotype derives from the coexistence of the 3 variants of these 2 genes, likely having an additive effect on neutrophilic inflammation.

This report highlights the importance of genetic screening for AIDs in patients with unexplained periodic fever syndromes, to avoid misdiagnosis and improper treatment. Further research is required to clarify the genotype-phenotype correlation in these diseases.

## Conflicts of interest

None disclosed.
